# Where Do Neurodevelopmental Disorders Go? Casting the Eye Away from Childhood towards Adulthood

**DOI:** 10.3390/healthcare11071015

**Published:** 2023-04-02

**Authors:** Giulia Antolini, Marco Colizzi

**Affiliations:** 1 Child and Adolescent Neuropsychiatry Unit, Maternal-Child Integrated Care Department, Integrated University Hospital of Verona, 37126 Verona, Italy; 2 Unit of Psychiatry, Department of Medicine (DAME), University of Udine, 33100 Udine, Italy; 3 Department of Psychosis Studies, Institute of Psychiatry, Psychology and Neuroscience, King’s College London, London SE5 8AF, UK

**Keywords:** neurodevelopment, autism, ADHD, specific learning disorders, intellectual disability, Tourette syndrome, neurodevelopmental continuum

## Abstract

Neurodevelopmental disorders (NDDs) encompass a group of complex conditions with onset during the early developmental period. Such disorders are frequently associated with a number of neuropsychiatric features, the most prevalent ones being autism spectrum disorder, attention-deficit/hyperactivity disorder, intellectual disability, communication and specific learning disorders, and motor disorders. These conditions are characterized by wide genetic and clinical variability, and although they were previously conceptualized as childhood-limited disorders, NDDs are progressively being recognized as persistent conditions with a potentially relevant impact on the quality of life and overall functioning during adult life. In addition, emerging evidence seems to point towards the hypothesis of a neurodevelopmental continuum, according to which NNDs could portray different time-dependent outcomes, depending on the severity of the altered brain development. Despite representing lifelong phenotypes, they are often not promptly identified and/or managed in adulthood. In this regard, specific guidelines on clinical and therapeutic approaches for these conditions have not yet been delineated. In this view, future research investigations should be encouraged to broaden available knowledge, characterize the clinical course of NDDs across an individual’s lifespan, and better understand the patterns of aging-related concerns in adults with an NDD diagnosis. Additionally, considering the difficulties many young adults encounter while transitioning from childhood to adult mental health services, new, specific programs should be developed and existing programs should be implemented to improve the transition process and for the management of NDDs in adulthood.

## 1. Laying the Groundwork

The term neurodevelopmental disorder (NDD) applies to a wide range of complex conditions that are characterized by a disruption in the chain of events that lead to normal brain development, with onset during the developmental period and consequent impairments in the individual’s global functioning [1,2,3]. According to the Diagnostic and Statistical Manual of Mental Disorders, Fifth Edition (DSM-5), NDDs comprise autism spectrum disorder (ASD), attention-deficit/hyperactivity disorder (ADHD), intellectual disability (ID), communication disorders, specific learning disorders (SLDs), and neurodevelopmental motor disorders, including tic disorders and Tourette syndrome (TS) (Table 1) [4]. Evidence suggests that communication disorders, in addition to specific learning disorders and developmental coordination disorder, are the most frequently encountered neurodevelopmental conditions, with worldwide prevalence rates estimated to be around 5–10%, 5%, and 5–8%, respectively. The estimated worldwide prevalence of ADHD is around 5%; ASD prevalence rates appear to be around 1.5% worldwide; ID seems to affect 1–3% of the worldwide population; and the estimated prevalence of TS is around 1% [5,6,7,8,9,10,11,12].

**Figure 1:**
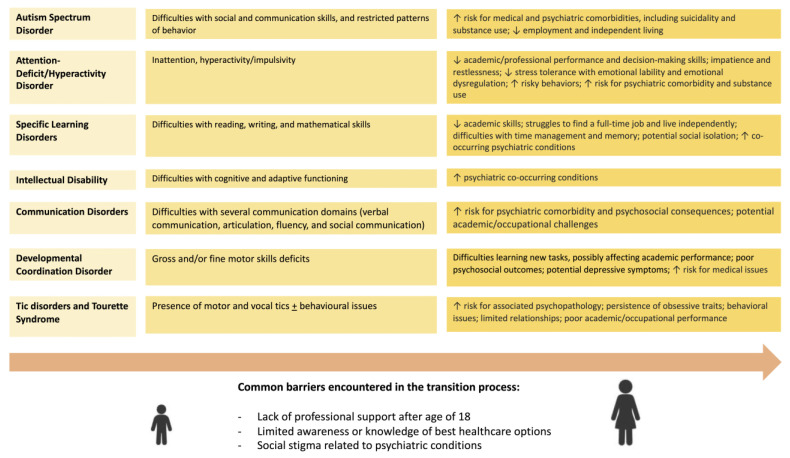
Graphical summary of the main clinical features and challenges encountered during adulthood for neurodevelopmental disorders.

NDDs have been shown to be characterized by a multi-factorial etiology and high clinical variability, with the commonality of onset during childhood. Both genetic and environmental factors are involved in the genesis of NDDs. They appear to be highly heritable, with a conspicuous number of genes identified as having a role in the etiology of these conditions and single major causes being rare [3,13,14,15]. Additionally, several environmental factors might be implicated in the origin of NDDs and may increase their incidence in genetically predisposed individuals; such factors include advanced maternal age, infections, and other medical conditions (e.g., obesity, gestational hypertension, and diabetes) in pregnancy, substance exposure and medication use during pregnancy, maternal psychological stress and postnatal depression, inborn metabolic errors, preterm birth, post-natal exposure to toxic substances, and neonatal problems (e.g., early natal infections, neonatal hypoxic-ischemic events, early development brain injuries, or neonatal deficiencies) [5,16,17,18,19,20,21,22,23]. Despite undergoing maturational changes over the years, potentially accounting for the manifestation of improvement with age, the natural course of an NDD is typically steady. As a consequence, the persistence of the condition into adulthood is not unlikely, and new, co-occurring disturbances may also emerge (Figure 1) [24,25,26,27,28,29,30].

## 2. A Life Course Perspective

In the past, many of the aforementioned NDDs were considered childhood-limited conditions [3]. Nonetheless, follow-up studies have shown that these disorders tend to persist across the lifespan, with variable outcomes and even changing phenotypes, having relevant implications for adult mental health services. At present, evidence for the existence of modifiable factors that optimize NDD outcome during adulthood is limited. There is a need for a thorough characterization of NDD across different ages and to adopt new and more flexible approaches to diagnosis and management. Some authors have proposed a model which envisions NDDs and other adult psychiatric conditions as lying on a neurodevelopmental continuum and not as separate entities. In this light, the complex clinical phenotypes, changing natural course across the lifespan, and clinical overlap that characterize NDDs represent a relevant area for future research studies [2,19].

### 2.1. Autism Spectrum Disorder

Autism spectrum disorder (ASD) is characterized by enduring difficulties in social and communication skills, as well as restricted patterns of behavior, interests, or activities [4]. In recent years, an increase in the prevalence of ASD has been registered and may rely on a number of factors, including an increased awareness in clinical practice and among families and teachers and changes in the diagnostic process, with current criteria being more inclusive with respect to previous years [31]. Regarding gender differences, ASD seems to be more prevalent in males; however, this may not reflect a real difference but rather a sex/gender bias, as diagnostic criteria seem to be more focused on male features [10].

ASD is usually identified during early childhood; however, in people with high levels of function and less severe manifestations, symptoms may sometimes become evident later in life, when social demands outpace the subject’s capability to deal with them [4]. Currently, there are no specific criteria to diagnose ASD in adult life, and undiagnosed adults may run into emotional and functional difficulties that relate to both the condition itself and to the fact that their struggles can be perceived as poorly understood and not adequately supported [8,9]. Thus, based on both historical information and actual presentation, early identification and diagnosis appear crucial [32]. Although manifestations may improve and change over the years, the disorder is a lifelong condition that persists into adult life and can affect the individual’s global functioning during the entire life course [33].

People diagnosed with ASD may have to deal with a variety of challenges when transitioning to adulthood; in this view, it was shown that only 21% of young adults with ASD had access to healthcare transition services, and 14% had the chance to discuss transitioning from pediatric to adult services with their childhood neuropsychiatrist [32,34]. Among the aforementioned challenges, there is an increased risk for medical comorbidities, which may be more common in individuals with ASD compared to the general population. These include seizures, gastrointestinal disorders, metabolic and cardiovascular abnormalities, sensory impairments, sleep disorders, and other mental conditions [32,33,35,36,37,38]. These health problems must be properly addressed to promote global functioning and health. Mental health comorbidities are particularly burdensome due to both symptom overlap and the impact of ASD’s core features on their management. Psychiatric conditions seem to be more frequent in individuals with ASD compared to the general population and subjects with other developmental disabilities [32]. These include mood disorders, anxiety, personality disorders, bipolar disorder, obsessive–compulsive disorder, schizophrenia, substance disorders, and other NDDs such as attention-deficit/hyperactivity disorder (ADHD) and intellectual disability (ID) [31,32,35,39,40]. In a study involving a sample of 52 young adults with ASD, Kraper et al. found that lower adaptive skills may be associated with more severe anxiety, depressive traits, and social difficulties [41]. Mood (especially major depression and dysthymia) and anxiety (in particular, general and social anxiety disorder and panic attacks) disorders are the most frequently observed mental health comorbidities in ASD subjects, with prevalence rates of 50–70% and 30%, respectively [32,42,43,44,45]. Suicidality also represents a relevant concern for young adults with ASD and, for this reason, it is recommended that this patient population is screened for suicidal ideation in addition to other mental-health related issues [32,46,47]. Furthermore, an increase in premature mortality rates has been observed in individuals with ASD, mainly following neurological disorders or congenital abnormalities [48,49,50]. Overall, it appears that such risk can be 2 to 10 times higher compared to the general population, independent of gender and a high- or low-functioning ASD phenotype [51,52,53]. Elevated mortality rates may be secondary to a number of causes, including nervous system conditions (e.g., epilepsy), chronic medical conditions (e.g., heart disease, respiratory disease, and cancer), accidents, and health issues due to the side effects of pharmacotherapy [48,50,54]. It should also be considered that as previously mentioned, suicidal ideation and suicide attempts may be present, especially in those with high-functioning ASD who frequently report comorbid mental health conditions and psychological vulnerability [13,50,55,56,57,58]. In addition, such individuals may lack protective factors that would decrease the risk of death by suicide (e.g., a supportive social network or good coping skills). Furthermore, difficulties with social interaction might affect their ability to seek and find the help they need [50,59,60,61]. The prevalence of substance use disorders ranges from 16% to 30% in subjects with ASD; such rates could be due to self-medication for psychiatric symptoms or to the need to deal with social impairments [56,62,63,64]. In addition, impairments in working memory and executive functioning may be present and persist into adult life [65].

Concerning education and employment, enrolling in tertiary education or employment may represent a challenge for young adults with ASD [66,67]. Studies show low levels of college attendance, and even students with ASD who present with good global intellectual functioning often do not obtain satisfying academic results and are at risk of dropping out of school [35,68]. Furthermore, evidence indicates that only one third of adults with ASD achieve paid employment [32,67,69]. Additionally, a considerable number of employed individuals may work part-time or not occupy a permanent position; occasional employment in hospitality, catering, and teaching may happen, as well as in scientific or engineering fields, which tend to be seen as areas in which people with ASD do well [32,70,71]. Factors which seem to be associated with an increased likelihood of employment include a high educational level, the presence of a strong social support network, and good intellectual functioning. On the contrary, subjects who report more severe ASD manifestations, language/cognitive impairment, and associated medical/psychiatric conditions are less likely to become employed [72].

The ability to fully live outside the parental home represents another indicator of a successful transition to adult life. Although future perspectives for people with ASD seem to be slowly improving owing to earlier recognition and more fruitful interventions, most of them still do not live an independent life [31,35,73,74,75,76]. According to the available literature, about 10–33% of adults with ASD require very substantial support in line with the DSM-5 classification; in addition, most individuals with comorbid ID may acquire a certain degree of autonomy in their daily lives but will still require support. Only a small percentage of adults are able to live independently without remaining in their parental home into adulthood [29,31,65].

As previously highlighted, ASD is characterized by considerably variable outcomes during adulthood, which are influenced by potential medical and psychiatric co-occurring conditions that must be adequately addressed to reduce their burden. A few prognostic predictors are known, namely, global intellectual functioning and communication skills; however, studies on the long-term effects of the most relevant interventions, both cognitive–behavioral and pharmacological interventions, are still lacking. Overall, favoring social support and inclusion may represent an important way to improve future perspectives for subjects with ASD and help them with the development of peer relationships.

### 2.2. Attention-Deficit/Hyperactivity Disorder

Although it was considered a disorder of childhood and adolescence for a long time, attention-deficit/hyperactivity disorder (ADHD) persists in adult life, with relevant impact on both professional and social functioning [77]. The DSM-5 defines three ADHD subtypes: (i) combined ADHD (presenting with both inattentive symptoms and hyperactivity/impulsivity), (ii) predominantly inattentive ADHD, and (iii) predominantly hyperactive/impulsive ADHD. Additionally, in order to make a diagnosis, several symptoms should be present in at least two settings, last at least 6 months, and interfere with the individual’s social and academic/professional functioning [4]. A considerable percentage of individuals receive a diagnosis after adolescence: the failure to identify core manifestations of ADHD with consequent misdiagnoses or the development of good coping abilities could be two of the reasons for diagnostic delay [77]. During adulthood, the clinical picture of ADHD may still involve the same core symptoms and according to the DSM-5, adults with ADHD may present with a predominantly hyperactive/impulsive or inattentive type or a combination of both [4,78]. However, manifestations may evolve over the years, and some differences can be observed: inattentive manifestations may be covered up by obsessive-like or anxious traits, and hyperactivity/impulsivity is often less pronounced when compared to childhood [9,77,78,79]. Additionally, the described symptomatology may be subtle, resulting in a functional impairment which may not be perceived by the individual [78]. This explains why the assessment of ADHD during adulthood requires a careful evaluation of the clinical picture as a whole and must take into account family impressions in addition to self-reports.

The persistence of ADHD manifestation in adult life can result in a number of relevant consequences that affect personal, professional, and relational life goals [9,77]. Distractibility and the inability to maintain focus may lead to poor academic or professional performance; furthermore, decision-making skills are commonly also hindered, often as part of a general executive functioning impairment, resulting in planning difficulties and inaccuracies, with consequent recurrent job changes or even unemployment. Instead, hyperactive presentations in adulthood often manifest as impatience and restlessness, with problems in waiting for one’s turn during conversations and a failure to respect interpersonal boundaries. This may also be associated with reduced stress tolerance, resulting in emotional lability and aggressive behavior [77,78,80]. Impulsivity is another dimension that is frequently present in ADHD that is often described as an aversion to delay during adulthood; it is more frequently verbal, and in addition to difficulties with sustaining attention, can lead to professional issues and risky behaviors (e.g., job-related or driving accidents or substance use). Emotional dysregulation is also commonly observed in people with ADHD, characterized by irritability and emotional outbursts, with a significant impact on social interactions and family care. Some studies have shown that adults affected by ADHD often report a lower educational degree, lower likelihood of becoming employed, and unstable familial relationships when compared to the general population [9,77,78,81,82,83,84].

Additionally, ADHD is frequently associated with co-morbid mental-health-related conditions, which can make the diagnostic process even more complex [77,85,86,87,88]. Co-existence with other psychiatric disorders appears to be as common as approximately 65% of children and 75% of adults with ADHD; such disorders include anxiety disorders, mood disorders, conduct and personality disorders (mainly antisocial and borderline personality disorders), and other NDDs (e.g., ASD and specific learning disabilities) [9,77,88,89,90,91]. Adults with ADHD are frequently exposed to stress, which is mainly linked to relational and professional difficulties, and consequent low self-esteem; as a result, major depressive disorder can be noted in about 20% of people with ADHD and can lead to an increased suicide risk [77,92,93]. Thus, ADHD is sometimes associated with suicidal behaviors, which are more common in presence of a mood disorder and impulsivity [77,94]. Manifestations of anxiety can be observed in almost one half of adults with ADHD and can be of different types, with the most frequently encountered types being social anxiety, panic disorder, and generalized anxiety disorder [77,92]. Additionally, in individuals presenting with severe impulsive behavior and limited tolerance to frustration, conduct disorders are a potential comorbidity in adult life, exacerbating the underlying disorder’s manifestations and contributing to the general functional impairment [9,78]. Substance use disorders represent another concern in adults with ADHD; these include alcohol and tobacco use, as well as cannabis use and other addictions. They can be seen as a consequence of emotional dysregulation or as an attempt at self-medication. Furthermore, sleep disorders (especially insomnia) are observed in approximately 60–80% of subjects with ADHD and often contribute to the exacerbation of symptoms [95,96,97,98,99].

ADHD itself and co-occurring conditions can result in significant functional impairment, with serious consequences in different life domains and a limited quality of life. Such considerations underline the importance of adequate assessment and management of the disorder, even in adult age. Once a diagnosis is reached, a thorough evaluation of the condition’s severity, as well as of the presence of potential comorbid medical and/or psychiatric issues, should be conducted in order to provide an adequate level of personalized care [77]. The management of ADHD during adulthood should aim at limiting unfavorable clinical manifestations and associated conditions, as well as their overall impact on the individual’s life. In this light, the mainstay of treatment for adult ADHD is represented by a combination of non-pharmacological and pharmacological interventions: non-pharmacological approaches include cognitive–behavioral therapy, psychoeducation, and psychological therapies; pharmacological treatments are instead mainly based on the use of psychostimulants, such as methylphenidate [77,100,101,102]. These strategies complement each other, with the medications mostly targeting core manifestations of ADHD and the non-pharmacological treatments reducing functional impairment. Overall, the clinical picture associated with ADHD undergoes relevant changes over time, and although identification of the condition in adults has recently increased, challenges still exist and mostly concern the transition process and access to adult services. In this light, future research investigations should focus more on transition age, and better training could be offered to general practitioners and psychiatry specialists in order to be able to diagnose ADHD in adults and ensure that they receive an adequate level of care and support.

### 2.3. Specific Learning Disorders

Specific learning disorders (SLDs) are a heterogeneous group of NDDs that are characterized by difficulties in reading (dyslexia), writing (dysgraphia and dysorthography), and/or mathematical skills (dyscalculia) in the presence of an intelligence quotient (IQ) that falls within the normal range and which are not better accounted for by an uncorrected auditory or visual acuity [4]. Prevalence rates are estimated to be around 5% worldwide [4,103]. SLDs are generally identified during early school years, with students reporting a learning profile that does not reflect their actual chronological and mental age, although in some cases, issues may emerge later. Additionally, symptoms may change over the years, and in a few cases, the concomitant presence of other disorders may make the diagnostic process more complex [103,104].

The majority of available studies have focused on children; however, it is now well-known that although they may improve with adequate early interventions, SLDs tend to persist into adult ages, with potential implications for daily functioning in several life domains [104]. During adulthood, these conditions may have an impact on the academic/work performance, as well as on the emotional and social spheres [105]. In particular, for individuals with SLDs, there is a risk for lower academic skills compared to their peers, which may interfere with professional performance or everyday life activities [103,104]. These subjects may strive to find or maintain employment. According to a to research study conducted in the UK, reading difficulties and limited mathematical skills may represent an obstacle to employment; furthermore, men and women frequently experience unemployment or struggle to find a full-time job and are more commonly occupied in manual work with low chances of promotion and a low paycheck [106]. The living situation of adult people with SLDs can be affected as well. Many of them may feel more confident in a well-known environment and may therefore not be able to fully live outside their parental home. In this regard, the additional uncertainties about finding a permanent job may lead to these subjects being reluctant to start their own families [103]. They may also struggle with time management, memory, and the ability to maintain focus during a conversation or a task [103,107]. As a result, they often feel socially isolated and have low self-esteem [103]. Social relationships for SLDs individuals are usually limited, in addition to their overall quality of life, in terms of global functioning, autonomy, and decision-making skills [104,108]. Another issue is represented by co-occurring psychiatric comorbidities, as it is not uncommon to find anxiety disorders (including somatic complaints and panic attacks), mood disorders (especially depression), personality disorders (e.g., emotionally unstable (borderline) personality disorder, dependent and anxious/avoidant personality disorder, and schizoid/anancastic personality disorder), and age-related conditions in this patient population [103,104,109,110].

Adults with SLDs may show different degrees of severity and may consequently demonstrate a wide range of functioning levels. They may report the persistence of spelling difficulties, slow reading, or issues with mathematical problem solving; thus, they may avoid engaging in activities requiring good reading, writing, or arithmetic skills, or the use of alternative approaches to access print, potentially with consequent socio-emotional distress [4,104]. Furthermore, the different developmental backgrounds, intersubjective characteristics, and co-existing conditions point out the extensive heterogeneity that characterizes the SLD continuum into adulthood. Unfortunately, the manifestations of SLDs during adult years are some of the least-examined areas, with a limited number of studies available on emotional, social, professional, and daily living outcomes. Thus, further evidence is needed to capture the complexity of these conditions and guide practice and research.

### 2.4. Intellectual Disability

Another neurodevelopmental condition characterized by a paucity of evidence-based studies during adult life is intellectual disability (ID). ID is characterized by limited cognitive and adaptive functioning. The onset is typically during the childhood period; however, it is a lifelong condition with potential consequences later in life. It affects approximately 1–3% of the worldwide population, with higher prevalence rates in developing countries, probably secondary to an increased number of births and limited access to appropriate healthcare services [11,12,111].

Presently, increasing percentages of individuals with a diagnosis of ID are living into adult age [112]. The ability to establish a proper diagnosis allows these patients to access adequate healthcare services. The diagnostic workup should comprise a detailed family and personal history, clinical assessment, and standardized psychological testing, which may be helpful in defining the individual’s cognitive and adaptive functioning profiles. Since it is important to assess the development of symptoms during childhood, it may be worth involving family members or caregivers in the diagnostic process whenever possible [4,23,113].

Epidemiological studies show a considerable number of adult subjects with ID reporting concomitant mental health related issues; these reports occur more frequently with respect to the general population. In addition, psychiatric comorbidities seem to increase with the severity of the ID. The most common conditions associated with ID are the following: behavioral disorders, mood disorders and anxiety, psychotic disorders, and other NDDs such as ASD, among others [9,114,115,116]. Conduct disorders appear to be among the most frequently encountered issues among this patient population; they are more common in those with a mild form of ID in which the increasing awareness of their functional difficulties compared to their peers and frequent underachievement in the professional and social fields may lead to feelings of inadequacy, low self-esteem, and anger, which may translate into emotional outbursts and aggressive behaviors [9]. Mood disorders represent another common condition in adults with ID. People with a milder type of ID may present with depressive traits that are similar to those observed in the general population; instead, more severely impaired subjects could manifest a low mood with symptoms of anxiety, agitation, and disruptive behaviors [117]. Anxiety can be present in up to 30% of individuals with ID, who may show both physical and psychological manifestations. The most frequently noted anxiety disorders include generalized anxiety disorders, panic disorder, obsessive–compulsive manifestations, and specific phobias (e.g., social phobia). Stress factors and/or associated depressive symptoms could worsen anxiety traits. Psychotic disorders and NDDs are also frequently correlated with ID, and sometimes it may be challenging to distinguish between the conditions, particularly in their most severe forms [9,114].

When a child who has been diagnosed with ID becomes an adult, the challenges they must face in meeting their needs may increase. This may be due to no longer having access to services specifically designed for children and/or no longer being surrounded by family members who can support and advocate for them. Thankfully, chances of ameliorating the quality of life for adults with ID are gradually increasing at present. Some community-based services can provide help in terms of daily living activities, providing a safe place to live, and providing programs based on maintaining as much independence as possible. Additionally, based on their abilities and global adaptive functioning, adults with ID may be able to live independently, requiring some degree of support. Common challenges include communication difficulties, public policy, and physical issues, which must be taken into consideration and carefully addressed [113]. Overall, there seems to be increasing awareness of the needs of people with ID as they move into adulthood. Thus, the potentially higher number of patients who are brought to medical attention as adults should be offered an appropriate diagnostic workup, as being able to receive a formal diagnosis can be of help in accessing adequate healthcare services that are tailored to the needs of each individual.

### 2.5. Communication Disorders

Communication disorders involve deficits in language, speech, and communication. They usually develop during childhood and are not better explained by other genetic or medical conditions [4].

*Language disorder* is defined by persistent difficulties in language acquisition and use, which may become evident across different forms of communication, including written, spoken communication, or even sign language [4]. It is secondary to deficits in the production or comprehension of language and generally influences grammar and vocabulary, limiting conversational skills [4]. Estimates suggest that approximately 7.4% of kindergarten children report some form of language disorder. Overall, expressive difficulties seem to be more common than receptive ones [118]. The clinical manifestations of this condition may vary according to age and severity: its recognition may gradually occur during the early developmental period, and children can show variable rates of language development [119]. Additionally, language disorder does not always face spontaneous resolution: it is likely to persist during adulthood, especially when diagnosed in children aged 4 years or older, with potential social consequences and a risk of peer victimization [4]. Furthermore, language disorder may co-occur with other NDDs, including specific learning disorder, ADHD (which appears to be the most common comorbidity), ASD, intellectual disability, motor and coordination disorders, social (pragmatic) communication disorder, speech sound disorder, and enuresis [4,119,120]. In addition, the emotional stress the combination of these conditions may create can lead to social withdrawal and adjustment disorders [119]. Evidence also shows that individuals with communication disorders may be four times more likely to report a psychiatric condition as well [121]. Further studies suggest that co-occurring psychiatric illness may exacerbate communication difficulties. Additionally, higher rates of anxiety disorders, disruptive behaviors, oppositional defiant and conduct disorders have been observed in subjects with a language disorder, resulting in increased functional impairment [119]. Thus, early identification and diagnosis based on both family history and actual presentation appear to be fundamental in implementing adequate interventions in an attempt to avoid persistence of the condition into adulthood.

*Speech sound disorder (SSD)* is characterized by difficulties with speech sound production which are not appropriate to an individual’s developmental stage and cannot be ascribed to another genetic or medical condition [4]. Usually, the onset of SSD occurs during early development, and the prevalence rate is estimated to be around 10% in young children, falling to 5% at about 6–7 years of age [119]. The clinical course of this condition appears more promising when compared to other communication disorders: milder cases may undergo spontaneous resolution, particularly if the subject does not face psychosocial consequences, and the majority of cases respond to adequate interventions and improve with time; however, in the case that the speech sound disorder is associated with a language disorder or an SLD, the prognosis is poorer, and the condition can persist into adolescence and adulthood [4,119]. Among potential comorbidities, subjects with SSD may report higher rates of language disorders as well as ADHD, and are consequently more likely to experience social withdrawal and discrimination, with an increased risk for mental-health-related issues in the longer term. Thus, early recognition appears crucial.

*Childhood-onset fluency disorder (stuttering) (COFD)* is one of the most commonly identified speech disorders and is defined by difficulties with speech fluency and time patterning, with proper diagnosis being made when difficulties are in excess of what is expected for the subject’s age [4]. It occurs in about 1% of children 10 years old and younger, whereas the prevalence slightly falls to 0.8% during adolescence [119]. It usually demonstrates a gradual onset, making its appearance during the early phases of development, and can wax and wane during childhood, with different degrees of severity [119]. Additionally, it may be more evident when the subject feels anxious or stressed and varies over time. Most cases, especially in females, recover to a major extent before or during adolescence. However, the persistence of symptoms at 8 years of age can be predictive of the endurance of the condition in later years [119]. Thus, COFD can be present even during adulthood and can impact the individual’s social and professional functioning. In particular, subjects with this condition can experience anxiety disorders (especially social anxiety disorder) and impairments with social communication, leading to the avoidance of social interactions [119]. In addition, the disorder can co-occur with other NDDs, such as ADHD, intellectual disability, ASD, SLDs, language disorder, speech sound disorder, and epilepsy, making the diagnostic process more challenging [4].

*Social (pragmatic) communication disorder (SCD)*. Individuals with SCD show persistent difficulties with using both verbal and nonverbal communication: they may report issues with social reciprocity and relationships, with understanding, taking part in social communication with peers, the appropriate use of communication in social settings, and adapting their communication style to their listener, and may not always show insight into their difficulties [4,119]. Such a condition arises during childhood and may involve spoken as well as written language and sign language [119]. Clinical manifestations may be evident at different times, and the clinical course is variable: some individuals significantly improve with time, while pragmatic difficulties in others persist into adulthood (especially in individuals whose symptoms appeared early), resulting in persistent impairment in social functioning and low performance in other associated skills (e.g., reading comprehension and written expression), with consequent academic and occupational challenges [4,119].

Overall, communication disorders span several domains of communication, and their evaluation and subsequent management requires a multidisciplinary team in order to implement the most appropriate intervention for an early resolution of the condition, to provide support to the individual and his/her family, and to promptly identify psychiatric comorbidity, which might negatively impact the long-term evolution of the disorder [119].

### 2.6. Motor Disorders

#### 2.6.1. Developmental Coordination Disorder

Developmental coordination disorder (DCD) is characterized by difficulties with motor coordination that significantly interfere with daily life activities and social and academic functioning [4]. Such a condition has its onset during the early developmental period, although it is generally not diagnosed before the age of 5 since there might be an appreciable variation in the age of motor skill acquisition, or the motor delay may not have fully appeared [4,122]. The worldwide prevalence is estimated to range from 5% to 8% in school-aged children, with males seemingly more affected than females [4,122,123,124,125].

It is not uncommon to find DCD associated with other NDDs, the most frequent being ADHD, with recent studies suggesting a potential genetic link between the two conditions [125,126,127,128,129,130,131]. Additionally, learning disabilities, communication disorders, specific learning disorders (especially those involving reading and writing), and ASD and conduct disorder may also co-occur with DCD [4,122,132,133,134,135]. The presence of comorbidities may make the diagnostic process more challenging and affect the accomplishment of daily life activities to a greater extent. As outlined in Criterion B of the DSM-5, the presence of motor coordination difficulties must significantly impact daily living activities and academic performance [4]. Those with this condition may report issues with academic-related tasks, with consequent poor school outcomes despite an average IQ [122,136]. Their difficulties with motor skills may also interfere with self-care (e.g., dressing, tying shoelaces, or using a knife and fork) and leisure participation, with secondary mental health and emotional concerns including low self-esteem, anxiety disorders, depressive traits, and emotional/behavioral disorders that might reduce their overall quality of life [122,123,137,138,139,140].

In the past, the common belief was that children presenting with DCD would improve in the longer term, outgrowing their motor difficulties [122]. However, evidence has shown that such difficulties may persist into adolescence and even adulthood, with outcomes outlying the motor domain to include emotional, behavioral, and mental-health-related issues [136,140,141,142,143]. Based on the personal experiences of families with children diagnosed with DCD, Missiuna et al. suggested a hypothesis according to which such condition could be characterized by a developmental continuum, with motor difficulties during early childhood, followed by academic, self-care, social, and emotional issues in adolescence [141]. Thus, although there may be some degree of improvement, difficulties with movement coordination are estimated to persist during adolescence in about 50% of subjects [4]. Furthermore, issues may be present even during adulthood, especially with learning new tasks, including difficulties with taking notes (which may influence professional performance), using tools, and driving. In addition, those reporting comorbid conditions may have poorer psychosocial outcomes and more depressive traits [138,144,145]. Furthermore, individuals with DCD seem to be at higher risk for obesity and cardiovascular conditions as they may show lower physical fitness compared to their peers [146,147].

Despite having to face several challenges, functional outcomes for subjects with DCD can be improved with interventions, which can be delivered by physical or occupational therapists, in addition to parents and teachers who may support their needs as they transition to adult life [122]. Additionally, individuals may learn to prefer occupations with less demand in the motor domain and to adopt compensatory strategies which allow for a positive outcome in adulthood [122,138].

#### 2.6.2. Tic Disorders and Tourette Syndrome

Tic disorders, including Tourette syndrome (TS), are heterogeneous disorders characterized by the occurrence of motor and vocal tics with onset during childhood and which persist for at least one year. Their worldwide prevalence is around 1% for children and 0.05% for adults [148,149,150,151,152]. Research on tic disorders, especially TS, has mainly focused on children and adolescents, with limited data available on the clinical course, global functioning, and quality of life in adults [153].

Tics usually follow a fluctuating course and make their first appearance around the age of six to seven years, often beginning with motor manifestations [148,154,155,156,157,158]. Subsequently, they may worsen for a few years before reaching a severity peak during the second decade of life [149]. The majority of individuals with such conditions experience a gradual improvement in tics over the years following adolescence, with one-third of individuals undergoing complete remission [149,153,158]. However, a relevant minority of subjects may have to deal with tic exacerbation and consequent issues regarding self-esteem, peer relationships, high anxiety rates, and poor school or work performance, with potential unemployment and low socio-economic status [149,159]. The main factors predicting the persistence of tics later in life and associated comorbidities encompass tic severity, depressive and stressful manifestations during early childhood, female gender, limited fine motor skills, and co-occurring medical and/or psychiatric conditions, including severe ADHD in childhood [160,161,162]. Some studies have even suggested a change in the body distribution of tics during adulthood, with a more axial involvement of motor manifestations and reduced phonic tics [163].

Additionally, one of the greatest concerns in subjects with tic disorders is represented by associated psychopathologies, including ADHD, obsessive–compulsive disorder (OCD), mood and anxiety disorders, conduct and personality disorders, substance use disorder, and sleep disturbances, all of which may significantly impact quality of life, social functioning, and academic/professional performance [8,149,160,161,164,165]. ADHD and OCD are the most commonly observed conditions, in addition to depressive manifestations [149]. The comorbidity of ADHD or OCD at the onset of a tic disorder is usually a predictor of a poor long-term outcome [166,167]. ADHD can be present in up to 60% of subjects with TS, with higher prevalence rates compared to the general population, and it may result in behavioral issues and greater global functional impairment than TS manifestations alone [148,154,167,168]. Furthermore, when tic disorders are associated with ADHD, symptoms of inattention and hyperactivity/impulsivity may precede the onset of tics; instead, in about 48% of patients, tics and ADHD manifestations are both present at the time the diagnosis is established [166,167]. The coexistence of ADHD and TS is associated with increased rates of anxiety and mood disorders, OCD, and personality and conduct disorders [154,169,170]. OCD can manifest itself at any time during the course of the underlying tic disease and can affect up to 60–90% of people with TS, interfering with global functioning and causing important distress [149,164]. OCD symptoms seem to reach a peak approximately two years after tics; additionally, obsessive traits generally tend to persist when moving into adulthood, whereas compulsive manifestations undergo a reduction over the years [8,161]. Furthermore, individuals with TS and comorbid OCD frequently present with obsessions about symmetry and violence, as well as compulsions to count, blink, or touch [149]. OCD in people with TS is also correlated with increased rates of mood and anxiety disorders, ADHD, personality disorders, and possibly even ASD [169,171]. As previously mentioned, mood disorders represent another concerning condition in patients with TS, with an estimated prevalence of 20% in epidemiologic and community studies [149]. In particular, depression can be associated with tic severity, behavioral issues, self-injury, and echo- or copro-phenomena [149,168]. Other psychiatric comorbidities possibly correlated to TS include personality disorders, sleep disturbances (especially nightmares, insomnia, and somnambulism), alcohol or drug abuse, and NDDs such as ASD, which seems to co-exist in approximately 13% of individuals and has some overlapping phenomenology [148,149].

Concerning the management of these disorders, educational and behavioral interventions are usually recommended as first-line approaches and are usually effective for subjects presenting with tic manifestations that do not impact global functioning. Instead, pharmacological therapies should be used in those whose symptoms lead to significant impairment of everyday life [159].

In summary, TS and tic disorders are multifaceted neuropsychiatric conditions which can profoundly impact the quality of life of children and adults. However, future research studies are needed in order to deepen the knowledge on the clinical course and longitudinal outcomes of such conditions across the lifespan, as well as their impact on global functioning.

## 3. Raising Concern about the Transition Process: The State of the Art and Future Directions

As previously mentioned, NDDs are most commonly identified during childhood but may represent lifelong conditions which persist into adult life, although their core symptoms may be subject to changes over the years [24,25,28,29,30]. Since they also present with complex and diverse clinical phenotypes, it appears to be of paramount importance to take into account the great overlap across these conditions when evaluating for them in clinical practice. In particular, it would be best to adopt a developmental view and flexible approach which considers the strong overlap and heterogeneity that characterize NDDs in terms of clinical presentations, treatment response, and outcomes. In this view, additional longitudinal research studies would be useful, reflecting what is detected in clinical practice to a greater extent and bridging child and adult life [172,173].

Now that longitudinal studies have shown that childhood psychopathological conditions continue into adulthood and may have a poor prognosis during adult ages, a critical aspect is represented by the transition from pediatric to adult healthcare services. This is a challenging process, characterized by a number of difficulties encountered by young adults after they reach the age boundary between services [174,175,176,177]. It is, however, a relatively common occurrence for young individuals presenting with lifelong mental-health-related conditions. Unfortunately, these individuals frequently find themselves without professional support after the age of 18 or are referred to adult services that are not adequately equipped to meet their specific needs [178,179,180]. A lack of engagement with adult services may result in adverse outcomes, namely, a loss of follow-up and reduced compliance with treatment, with potentially negative consequences in both the educational and social spheres [181,182,183]. In turn, poor engagement can follow inadequate preparation for the transition process while the adolescent patient is still under childhood neuropsychiatric care or unsatisfactory transition planning [184,185]. Thus, a healthy, planned, coordinated, patient-centered interaction between childhood neuropsychiatrists and adult healthcare professionals seems to be essential in promoting a smooth transition process and ensuring continuity of care [172].

Currently, individuals presenting with severe mental health conditions (e.g., psychosis) seem to be more likely to transition to adult healthcare services, whereas those with NDDs report more difficulties in the transition process [186,187]. For instance, studies from the U.K. and U.S. report that only about 15% of young adults diagnosed with ADHD experience an adequate transition process to adult mental health services; moreover, the availability of adult services is limited, as is the availability of specifically trained professionals [186,188,189]. A few studies performed in developing countries have confirmed that only a limited number of young adults engage with adult healthcare services. Patients and their families may not feel properly supported during the transition process, they may not know exactly what options are available for them, or they may refuse to seek help due to stigma surrounding psychiatric conditions [190,191]. Furthermore, the complex and changing clinical pictures of NDDs, with potentially associated medical and psychiatric co-occurring issues, may not follow the strict, known criteria for adult services, which already find themselves facing a high number of transitions [186,191].

One way to improve the outcomes could be represented by the introduction of transition programs [192]. These may include implementing a progressive transition process, adequately moving the management of a disorder to the adult team in a coordinated way that is comprehensive of updated treatment plans, screening for comorbid conditions, and involves families and/or caregivers and the patient’s family physician in the process. In this view, which is unfortunately not always possible, joint visits between the child and adult teams would be of paramount importance, as the more clinical information the childhood neuropsychiatrist is able to provide, the higher the chances are for an effective transition to the adult service [173,186,193,194,195,196]. Additionally, adopting a multidisciplinary program to handle the diverse issues young adults with NDDs may present could be ideal, and transition programs should focus on comorbidity treatment as well. Another aspect worth mentioning is the training of adult professionals in dealing with childhood-onset mental health conditions [173,197]. Luckily, it appears that things are now gradually changing. For instance, transition and counseling groups are emerging as young adults must often rely on themselves for daily life duties but may not have developed the capability to effectively navigate services and healthcare systems, thus requiring support. In this light, transition groups and the structured interventions they offer to young people and their families can be of help in the process of adjusting to independently living in a community [198]. Furthermore, some organizations have developed multidisciplinary teams to work in concert with children and adult mental health services to meet the needs of young adults, and online resources are available to support people moving from adolescence into adulthood [199,200].

Overall, many young people report a number of difficulties when transitioning to adult mental health services and are at risk of finding themselves without a proper referral or any type of support [186,194,201]. While transitioning from childhood to adult healthcare services, adolescents/young adults may go through a lack of continuous and adequate care [181,182,183]. Transitional times represent a critical period for those with lifelong mental health conditions when new issues may emerge; thus, paying attention to the transition process could even improve the general outcome [173]. Successful results include not only control over core symptoms but also improvements to the overall quality of life, psychological wellbeing, professional and educational outcomes, and global functioning level. Therefore, it seems important to develop and implement already existing transition programs to make adult healthcare services available, targeting the individual needs of adults presenting with NDDs [172].

## Figures and Tables

**Table 1:** Summary of core manifestations, prevalence rates, and life-course trajectory of the main neurodevelopmental disorders (NDDs).

NDD	Core Features	Prevalence Rates	Age at Onset	Evolution
Autism spectrum disorder	Qualitative impairment in social communication skills, with stereotyped and restrictive patterns of behavior.	Around 1.5%	Early developmental period	Lifelong; adults with autism spectrum disorder frequently have to face several challenges with respect to co-occurring medical and psychiatric conditions, education, work, and living situations.
Attention-deficit/hyperactivity disorder	Inattention, hyperactivity, and impulsivity.	5%	Before 12 y.o.	Some symptoms may persist into adulthood, although the clinical presentations may change with time and concomitant psychiatric issues may emerge.
Specific learning disorders	Significant deficits in basic writing, reading, or mathematical skills.	5%	Within the first years of elementary school	May improve with early, adequate interventions or may persist into adulthood.
Intellectual disability	Limited cognitive and adaptive functioning.	1–3%	Within the first 3 years of life	Lifelong; frequently associates with psychiatric comorbidities in adult age.
Communication disorders	- *Language disorder*: difficulties in language acquisition and use, secondary to deficits in language production or comprehension. - *Speech sound disorder*: Difficulties with speech sound production. - *Childhood-onset fluency disorder*: Difficulties with speech fluency and time patterning. - *Social (pragmatic) communication disorder*: Difficulties with social use of language and communication.	- *Language disorder*: around 10% - *Speech sound disorder:* around 5% - *Childhood-onset fluency disorder:* around 1% - *Social (Pragmatic) communication disorder:* still limited data	Early developmental period	- *Language disorder*: can improve with early, adequate interventions or may persist into adult life (especially if diagnosed in children ≥4 y.o.); it may have social consequences. - *Speech sound disorder*: frequently responds well to treatment; it may be lifelong when associated with a language disorder/specific learning disorder. - *Childhood-onset fluency disorder:* most cases recover from dysfluency; however, it may persist into adulthood. - *Social (Pragmatic) communication disorder:* variable outcomes; in some cases, difficulties persist into adult life.
Developmental coordination disorder	Difficulties with gross and/or fine motor skills, interfering with daily life activities	5–8% in children ages 5–11 y.o.	Early developmental period	Symptoms may improve; however, coordination difficulties may persist throughout adolescence and adulthood, along with issues in learning new tasks, which may affect professional performance.
Tic disorders	Involuntary, repetitive, sudden twitches, movements, or vocalizations (tics)	1%	Between 2 and 15 y.o. (average around 6 y.o.)	Tic severity improves after adolescence for most patients; tics may persist into adulthood in a relevant minority of subjects who may report low self-esteem, limited peer relationships, high anxiety rates, and poor school or work performance.

## Data Availability

Not applicable.
